# Responsiveness in DoC and individual variability

**DOI:** 10.3389/fnhum.2015.00270

**Published:** 2015-05-15

**Authors:** Walter G. Sannita

**Affiliations:** ^1^Department of Neuroscience, Ophthalmology and Genetics, University of GenovaGenova, Italy; ^2^Institute S. Anna – Research in Advanced Neuro-RehabilitationCrotone, Italy; ^3^Department of Psychiatry, State University of New York at Stony BrookStony Brook, NY, USA

**Keywords:** brain, physiology, disorder of consciousness, vegetative state, variability

Neuroimaging has shown the capability of the severely injured brain to retain high-level functional aspects across sensory modalities, language processing, and learning dynamics also in the *vegetative state/unresponsive wakefulness syndrome* (VS/UWS) (Monti et al., 2010a; Monti, 2012; Gibson et al., 2014; Gosseries et al., 2014). Regional brain activations suggesting awareness and cognition were reported in a small number of subjects (Monti et al., 2010a); whether these activations should be considered equivalent to consciousness or compatible with, but atypical for VS/UWS is a matter of debate (Bruno et al., 2010; Monti et al., 2010b; Bardin et al., 2011; Celesia, 2013). The residual functions were otherwise thought to result from intact but functionally disconnected cortical modules that do not necessarily give rise to phenomenal consciousness. The preservation of specific neural structures and available residual cognitive resources has been suggested to vary in these patients because of the heterogeneity of etiology and pathophysiology or extension and severity of brain damage (Gibson et al., 2014).

These findings have changed the scenario in raising questions about the underlying brain functions that may sustain consciousness (Monti et al., 2010b; Celesia, 2013). Functional neuroimaging is now available only in selected centers and is mainly a research tool; diagnosis, monitoring of evolution, and early prognosis of VS/UWS still rest mostly on the clinical evaluation of responsiveness (Celesia, 2013). Several established indicators, however, have proven unstable over time also in single subjects (Bosco et al., 2010; Candelieri et al., 2011; Cortese et al., in press). For instance, the observation of a visual pursuit response (a marker of evolution from VS/UWS) varies over time and during the day in both VS/UWS and minimally conscious state when subjects are repeatedly tested (Candelieri et al., 2011; Cortese et al., in press). It is highest around 10.30 am and 3.00 pm (Cortese et al., in press) and higher in the morning compared to afternoon (Candelieri et al., 2011), with no response at post-prandial time. The probability of observing a visual pursuit response at least once over the course of a single day is around 30–40% (Candelieri et al., 2011; Cortese et al., in press), consistent with the reported rate of misdiagnosis between VS/UWS and the minimally conscious state (Andrews et al., 1996; Schnakers et al., 2009; Rosenbaum and Giacino, 2015).

Residual fragments of circadian/ultradian cycles asynchronous among DoC subjects are possible, but fluctuations of the brain functional state unrelated to circadian/ultradian cycles are observed in healthy humans and animals. These fluctuations correlate with several neuronal and non-neuronal biological parameters that vary within the physiological range depending on the momentary functional or homeostatic requirements and interact with each other for reference (Sannita, 2006). Examples are the modulation of electrophysiological brain signals by spontaneous or environment-related changes in temperature, metabolism (e.g., glucose, ammonia), blood flow, oxygen extraction, pO_2_, pCO_2_, blood availability of ferritin, hormones (thyroxine, sexual hormones, cortisol, steroids, ATCH), and neurohormonal interaction, light-dark regulation of serotonin, histamine, and dopamine/melatonin/vitamin B_12_ secretion and interaction, etc. These factors can individually or collectively account for unexplained or underestimated individual variability and may become clinically relevant on occasions, as can be the case with sex hormones modulating anesthesia or epileptic seizures for reference (Sannita, 2006).

Systematic studies of the end-effect of physiological neuronal or non-neuronal factors on responsiveness in DoC are still lacking. However, direct/indirect functional links between autonomic nervous control and the activity in brain structures involved in higher brain functions, including conscious processes, have been documented (Napadow et al., 2008). Accordingly, physiological descriptors of the autonomic nervous system functional state and sympathetic/parasympathetic balance have been found to correlate with, and predict with high accuracy the occurrence of clinical signs of responsiveness in VS/UWS (Riganello et al., 2013).

The clinical criteria in use to characterize these subjects and predict outcome should be reconsidered and include their variability over time. Multiple testing would reduce the risks of misclassification (Candelieri et al., 2011; Cortese et al., in press). The correlation with the functional state of the autonomic nervous system should be considered and monitoring should be extensive and focus also on non-neuronal factors. Reclassification of some patients or classes of patients may prove appropriate if based on systematic investigation. In general, variability over time of neuronal/non-neuronal parameters should be regarded as an independent variable *per se*. It is a potential source of bias, or it could qualify in many instances as *the* information of interest. In either case, it should be taken into proper account in research when planning/performing experiments in otherwise controlled conditions as well as in clinical care.

## Conflict of interest statement

The author declares that the research was conducted in the absence of any commercial or financial relationships that could be construed as a potential conflict of interest.
